# Expression of Telomere Repeat Binding Factor 1 and TRF2 in Prostate Cancer and Correlation with Clinical Parameters

**DOI:** 10.1155/2017/9764752

**Published:** 2017-07-20

**Authors:** Wei Chen, Yong Wang, Fei Li, Wei Lin, Yong Liang, Zhiwei Ma

**Affiliations:** ^1^Department of Urology, Zigong Fourth People's Hospital, Sichuan, China; ^2^Department of Pathology, Zigong Fourth People's Hospital, Sichuan, China; ^3^Department of Urology, Nanfang Hospital, Southern Medical University, Guangzhou, China; ^4^Department of Urology, Sichuan Academy of Medical Sciences and Sichuan Provincial People's Hospital, Chengdu, China

## Abstract

**Objective:**

The objective of this study was to investigate the expression of telomere repeat binding factor 1 (TRF1) and TRF2 in prostate cancer and their relationships with clinicopathological features.

**Methods:**

In total 50 prostate cancer tissues and paired benign prostate hyperplasia tissues were analyzed. The telomere-binding proteins TRF1 and TRF2 were measured using immunohistochemical method. Correlation analyses were used to evaluate the association between immunohistochemical score and clinical parameters.

**Results:**

The expression of TRF1 was significantly higher in prostate cancer tissue than in benign prostate hyperplasia tissue (*χ*^2^ = 62.69, *P* < 0.01). Elevated levels of TRF2 were observed in both prostate cancer and benign prostate hyperplasia tissue (*χ*^2^ = 1.13, *P* = 0.76). TRF1 expression was significantly positively correlated with surgical capsular invasion (Spearman's *r* = 0.43, *P* = 0.002), seminal vesicle invasion (Spearman's *r* = 0.35, *P* = 0.01), lymph nodes metastases (Spearman's *r* = 0.41, *P* = 0.003), total prostate specific antigen (*r* = 0.61, *P* < 0.05), and Gleason score (*r* = 0.47, *P* = 0.01). However, there were no significant statistical differences between prostate volume (*r* = 0.06, *P* = 0.75) and age (*r* = 0.14, *P* = 0.09).

**Conclusion:**

Both TRF1 and TRF2 were overexpressed in prostate cancer. There was no specificity of TRF2 in prostate cancer, while TRF1 may be associated with prostate cancer progression.

## 1. Introduction

Prostate cancer is the second common cancer of men in developed countries. It is also the third cause of cancer death in men, which is secondly to lung and bronchial carcinoma [1]. The prevalence of prostate cancer is gradually rising even though the diagnostic methods were rapidly improving. Therefore, it is necessary to further study the pathogenesis and diagnosis methods. Telomere is an important structure of chromosomes, which plays an important role in maintaining the stability of the chromosome [2]. There were different degrees of telomere loss or telomere unstable phenomenon in most tumors. Studies show there was different expression in cervical cancer and stomach cancer of telomere repeat binding factors 1 and 2 (TRF1 and TRF2), which maintained the stability of telomere. However, up to now, there was no study report about TRF1 and TRF2 expression in prostate cancer, as well as the relationship between the telomere repeat binding factor and clinical pathological variables [3, 4]. This study intended to detect the expression of TRF1 and TRF2 in prostate cancer tissue and discuss the correlation between clinical pathological indicators, so as to further clarify the molecular mechanisms of prostate cancer.

## 2. Materials and Methods 

### 2.1. Inclusion and Exclusion Criteria

The presented study was approved by the hospital ethics committee of Zigong Number 4 People's Hospital and all patients gave informed consent before operation. Prostate cancer tissue and paired benign prostate hyperplasia tissue were obtained from patients who underwent surgical resection or transrectal prostatic biopsy in Department of Urology from January 2015 to April 2016. The sample size in each group was 50 cases. The inclusion criteria were (1) patients with pathological diagnosis of prostate cancer or benign prostate hyperplasia and (2) no age restriction. The exclusion criteria were as follows: (1) patients with urinary tract infection or systemic infection; (2) metastatic prostate cancer; (3) pathological diagnosis confirming prostatic hyperplasia accompanied by suspicious prostate cancer cell. The ages of patients in this study were ranging from 51 to 76 years (median 62 years). Any preoperative treatment was not applied on each patient. All patients with prostate cancer were scored according to Gleason system.

### 2.2. Immunohistochemical Assay

All tissue specimens were confirmed by pathological examination. Specimens were immediately stored at −90 centigrade after excision. Before antigen-antibody reaction, the tissue was fixed with 4% paraformaldehyde for 15 min and then the 5 *μ*m slices were made on the paraffin slicing machine. All antigen-antibody reactions are performed in accordance with manufacturer's instructions. TRF1 and TRF2 primary monoclonal antibodies were obtained from Abcam Co. Ltd. (Cambridge, UK). The dilution multiple was set as 1 : 100.

### 2.3. Immunohistochemical Results Definition

Semiquantitative calculation was obtained to assess the immunohistochemical results. Brown granules were observed in positive reaction of TRF1 and TRF2 protein. Five independent regions were observed and scored at 400x magnification under microscope by two pathologists in each slice. The final score of each slice was calculated from the average value of 5 regions. Positive cells number in slice was scored using the three-point scale: no staining observed in slice is scored 0. While observed mild staining is scored as 1, observed moderate staining is scored as 2, and observed deep staining is scored as 3, positive cells rates were still scored using three-point scale: the proportion of positive cells was scored as 0 (absence of positive cells), 1 (<10% positive cells), 2 (11-50% positive cells), and 3 (>50% positive cells).

The final immunohistochemistry score was defined as positive cells number multiplied by positive cells rate. Negative was defined as 0–3 points, 3-4 points meant weak positive (+), 5–7 points meant moderate positive (+), and 8-9 points meant strong positive (+++).

### 2.4. Statistical Analysis

All statistical analyses were performed using SPSS version 20.0 (SPSS Inc., Chicago, IL, USA) to evaluate the significance of mentioned variables. Measurement data was expressed as mean and standard deviation. Chi-square test was obtained to evaluate categorical data. Two independent Student's *t*-tests were used to determine the different between groups. One-way analysis of variance was appropriate for data comparison between multigroups. Spearman correlation analysis was appropriate for evaluating the correlation between TRF1 and surgical capsule, seminal vesicle invasion, and lymphatic metastasis. *P* value < 0.05 was considered to be statistically significant.

## 3. Results

### 3.1. Basic Characteristic between Groups

A total of 50 prostate cancer cases and 50 benign prostate hyperplasia cases were included in this study. The mean age was 63.24 ± 13.92 years (range, 55~75) in prostate cancer group and 61.81 ± 10.24 years (range, 57~79) in benign prostate hyperplasia group, respectively. The body mass index (BMI) was 23.82 ± 5.27 kg/m^2^ in prostate cancer group and 22.54 ± 4.98 kg/m^2^ in benign prostate hyperplasia group, respectively. The total prostate specific antigen (TPSA) was 26.31 ± 8.19 ng/ml (range, 1.76~36.12 ng/ml) and 1.75 ± 0.24 ng/ml (0.00~3.19 ng/ml) in benign prostate hyperplasia group. The Gleason score was 6.19 ± 0.61 (range, 4~8) in prostate cancer group. In these cases, the Gleason score was higher than 7 for 32 patients. There were 36 cases with surgical capsular invasion, 34 cases with seminal vesicle invasion, and 25 patients with regional lymph node metastasis according by pathological diagnosis. Statistical differences were not detected in terms of age and BMI between groups (*P* < 0.05); see Table 1.

### 3.2. Expression of TRF1 and TRF2 in Prostate Cancer and BPH Tissue

**Figure 1:**
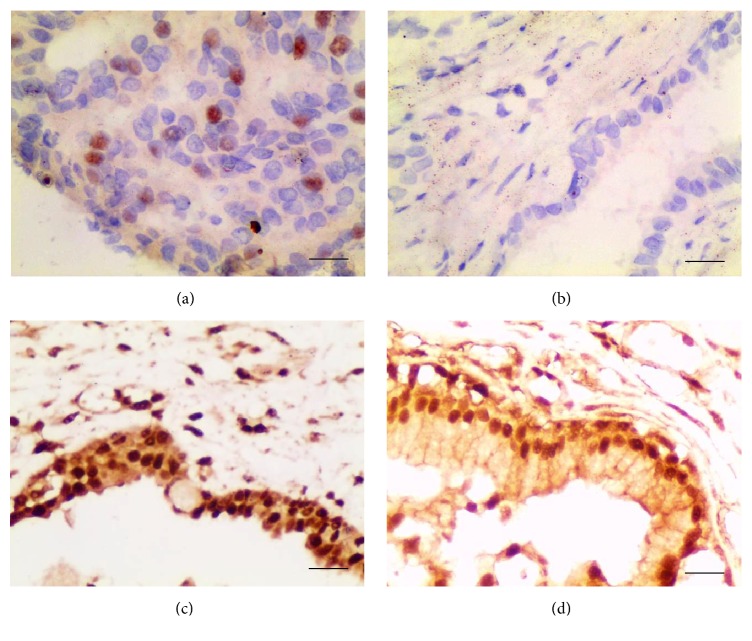
Expression of TRF1 and TRF2 in prostate cancer and benign prostate hyperplasia (×400; (a) TRF1 in prostate cancer; (b) TRF1 in benign prostate hyperplasia; (c) TRF2 in prostate cancer; (d) TRF2 in benign prostate hyperplasia).

The expressions of TRF1 and TRF2 in prostate cancer and BPH tissue are provided in Figure 1 and Table 2. Immunohistochemical results demonstrated that TRF1 protein was mainly expressed in the nucleus both in prostate cancer and in benign prostate hyperplasia tissues. However, the level of TRF1 in prostate cancer was significantly higher than that of benign prostate hyperplasia (*χ*^2^ = 62.69, *P* < 0.01). TRF2 protein was more highly expressed in both prostate cancer and benign prostate hyperplasia tissues. Both nucleus and cytoplasm could detect TRF2, which was mainly located in nucleus. There was no statistical significance between groups in terms of immunohistochemical staining score (*χ*^2^ = 1.13, *P* = 0.76).

### 3.3. Correlation of TRF1 and Clinical or Pathological Variables

The relations between the TRF1 stain and surgical capsular invasion, seminal vesicle invasion, and lymph node metastasis are provided in Table 3 and TPSA levels, Gleason score, prostate volume, and age are provided in Table 4. No significant relationships were found between TRF1 stain and patient's age and prostate volume (*P* = 0.72, *P* = 0.36). The TRF1 stain was stronger in patient with surgical capsular or seminal vesicle invasion (Spearman's *r* = 0.43, *P* = 0.002; Spearman's *r* = 0.35, *P* = 0.01, resp.). Patients with high level of TPSA always meet a strong stain of TRF1 (*F* = 5.61, *P* = 0.01). Staining for TRF1 was stronger in advance stage of Gleason score than in lower stage of Gleason score (*F* = 4.97, *P* = 0.03). Moreover, TRF1 was higher in tumors from patients with lymph nodes metastasis than in those without lymph nodes metastasis (Spearman's *r* = 0.41, *P* = 0.003).

## 4. Discussion

Studies have shown that telomere instability is one of the main causes of prostate cancer. Molecular biology studies suggest that the main function of telomere is to maintain the integrity of chromosomes. Telomere can prevent the destruction of chromosome structure caused by endogenous or exogenous factors. Telomere locates at the end of linear chromosomes and is composed of telomere-binding proteins and repeat TTAGGG sequence. It plays an important role in tumor development belonging to its cap-like structure nature [5].

5′ RNA primer guide model is essential for semiconservative DNA replication in cell division, which also exists in chromosome replication progress. However, literature reported that chromosome 3′ end, the template of DNA replication, usually cannot be completely copied, so that telomeres at the region cannot be corresponding fully replicated [6]. Generally speaking, the ends of chromosomes in normal cells cannot have endless growth. Cell division will be stopped and enter the stage of programmed cell death when the growth ended [7]. While chromosome semiconservative replication process is not shortened the telomeres and programmed cell death will be suppressed in most malignancies. Thereby consecutive proliferation of tumor cells can be detected. One of the reasons is that there is more highly active telomerase in these cells, which can promote the cell growth and telomere extending [8]. Sarek et al. demonstrated that tumor cell proliferation is regulated by different mechanisms other than telomerase regulation [9]. Research found that telomere-binding protein plays an important role in maintaining telomere stability and telomere length regulation [9–11].

Studies showed that the expression and biological function of telomere-binding protein are limited to telomeres, which mainly includes telomere repeat binding factor 1 (TRF1) and TRF2. The structure and function of TRF1 and TRF2 can antagonize or cooperate with each other in different tissues, so that component of the network maintained the ends of chromosomes stable by regulating telomere length and telomerase activity [12, 13]. The role of negative regulation of telomere terminus of TRF1 is mainly dependent on telomerase; its main function is to promote the T-loop formation located at telomere terminus. According to its role, TRF1 overexpression will shorten the telomere terminus length, thus contributing to tumor cells abnormal proliferation [14–16]. TRF2 does not depend on telomerase; the main role is to prevent telomere terminus fusion and maintain the genetic information and telomeres structure stable [17, 18].

In present study, we have evaluated the differential expressing of TRF1 and TRF2 in prostate cancer and benign prostatic hyperplasia by immunohistochemical method. We detected an elevated level of TRF1 and TRF2 in prostate cancer tissue. Overexpression of TRF2 has been found in both prostate cancer and benign prostatic hyperplasia, whereas there was no statistical difference between groups. Further analysis on the relationship between TRF1 and clinical and pathological indicators of prostate cancer found there were significant elevated levels of TRF1 in patients with surgical capsular invasion, seminal vesicle invasion, or lymph node metastasis and high level of TPSA and Gleason scores, while there was no significant correlation with prostate volume, prostate size, and age.

In summary, there was specific elevation of TRF1 expression in prostate cancer that could be a potential indicator to evaluate prognosis. The fact that the molecular mechanisms of TRF1 impact on the biological behavior of prostate cancer needs further research.

## Figures and Tables

**Table 1:** Basic characteristics between groups.

Indicator	PCa (*n* = 50)	BPH (*n* = 50)	*t*/*χ*^2^	*P*
Age (years old)	63.24 ± 13.92	61.81 ± 10.24	0.59	0.56
BMI (kg/m^2^)	23.82 ± 5.27	22.54 ± 4.98	1.25	0.21
TPSA	26.31 ± 8.19	1.75 ± 0.24	21.54	<0.001
Gleason score	6.19 ± 0.61	NA	NA	NA
Surgical capsular invasion	36	NA	NA	NA
Seminal vesicle invasion	34	NA	NA	NA
Regional lymph node metastasis	25	NA	NA	NA

PCa, prostate cancer; BPH, benign prostatic hyperplasia; BMI, body mass index; PSA, prostate specific antigen; NA, not available.

**Table 2:** Expression of TRF1 and TRF2 in prostate cancer and benign prostatic hyperplasia.

Protein	Pathology^*∗*^	−	+	++	+++	Positive ratio	*χ* ^2^	*P*
TRF1	PCa (*n* = 50)	6	8	31	5	88.0%	62.69	<0.01
	BPH (*n* = 50)	44	5	1	0	12.0%		
TRF2	PCa (*n* = 50)	8	7	17	18	84.0%	1.13	0.76
	BPH (*n* = 50)	11	9	14	16	78.0%		

^**∗**^PCa, prostate cancer; BPH, benign prostatic hyperplasia.

**Table 3:** Correlation between TRF1 and surgical capsular invasion, seminal vesicle invasion, and lymphatic metastasis.

		− (*n* = 6)	+ (*n* = 8)	++(*n* = 31)	+++ (*n* = 5)	*χ* ^2^	*P*
Surgical capsular invasion	Yes	2	4	25	5	9.46	0.02
	No	4	4	6	0		
Seminal vesicle invasion	Yes	1	5	24	4	8.97	0.03
	No	5	3	7	1		
Lymphatic metastasis	Yes	0	2	20	3	10.81	0.01
	No	6	6	11	2		

**Table 4:** Correlation between TRF1 and age, TPSA, Gleason score, and prostate volume.

	− (*n* = 6)	+ (*n* = 8)	++ (*n* = 31)	+++ (*n* = 5)	*F* ^*∗*^	*P*
TPSA	21.08 ± 4.89	26.19 ± 5.61	27.91 ± 9.68	29.64 ± 11.03	5.61	0.01
Gleason score	5.32 ± 0.84	5.86 ± 1.03	6.71 ± 0.48	8.61 ± 1.35	4.97	0.03
Prostate volume	78.32 ± 7.85	75.93 ± 5.46	75.69 ± 9.61	83.26 ± 5.73	0.93	0.36
Age	60.69 ± 12.67	61.37 ± 12.53	66.48 ± 9.52	63.29 ± 11.94	0.35	0.72

^*∗*^ *F*, variance of *F* value.
